# Supplementary material for: Are carbon emissions trading and green financial instruments synergistic? -Comprehensive quantitative research based on content analysis
Source: PLoS One. 2024 Mar 7;19(3):e0298601. doi: 10.1371/journal.pone.0298601 (PMC10919601; doi:10.1371/journal.pone.0298601)
Supplement: S1 Data — (ZIP) [file pone.0298601.s001.zip › Data/地级市Z_F工作报告.zip!/╣π╢1⁄2/╣π╓▌╩╨/└┤╫╘╣1⁄2╓┌║┼í╛┬φ┐╦╩2╛▌═°í┐_2015.docx]

各位代表：

　　现在，我代表广州市人民政府，向大会作工作报告，请予审议，并请市政协委员和其他列席人员提出意见。

　　一、2014年工作回顾

　　过去一年，面对经济下行压力加大的突出矛盾，我们认真贯彻党的十八大、十八届三中、四中全会和习近平总书记系列重要讲话精神，在市委正确领导下，在市人大、市政协监督支持下，坚持为民，用心做事，全力以赴稳增长、促改革、调结构、惠民生、增后劲，经济社会发展取得明显成效。

　　——经济运行总体平稳。地区生产总值1.67万亿元，增长8.6%。居民消费价格涨幅2.3%。城镇登记失业率2.26%。进出口总值1306亿美元，增长9.8%。广州第四次被评为福布斯中国大陆最佳商业城市第一名。

　　——质量效益持续提高。源于广州地区财政预算收入4834亿元，增长9.1%;地方一般公共预算收入1241.53亿元，增长8.7%(可比口径12.9%)。城乡居民可支配收入增长与经济增长基本同步，差距继续缩小。

　　——结构调整取得实效。三次产业比重为1.42︰33.56︰65.02。服务业比重持续提高，高技术产业、战略性新兴产业和新业态加速壮大。万元生产总值能耗下降3.62%，主要污染物排放量明显下降。

　　——社会事业蓬勃发展。科技、教育、文化、卫生、体育、治安等领域取得新进步，十件民生实事全面兑现，民生发展指数在省会城市和计划单列市中排名第一。

　　一年来的主要工作及特点：

　　一是有效应对经济下行压力，促进经济平稳较快增长。面对国内外复杂环境，坚持把发展作为第一要务，消费、投资、外需均衡增长，协同拉动经济稳中向好。

　　促进投资提速。发挥投资的关键作用，推进136个重点项目，完成投资总额1109亿元。举办“新广州·新商机”招商推介会，加大重大发展平台投资力度，鼓励吸引民间投资，促进投资稳定增长。固定资产投资4889.5亿元、增长14.5%，民间投资增长24.9%。

　　挖掘消费潜力。着力增强消费的拉动作用，推进重点商圈升级改造，加快国际会展中心、物流中心、采购中心建设，完善商贸功能。开展一系列专题商贸展贸活动，促进信息、文化、健康、旅游等新型消费，激发消费活力。旅游综合竞争力列全国副省级城市第一。社会消费品零售总额7697.85亿元，增长 12.5%。

　　开拓外需市场。着力改善外贸环境，自贸试验区申报成功，海关特殊监管区域建设有新进展，落实出口退免税401.1亿元。拓展国际市场，开拓 “21世纪海上丝绸之路”通道，鼓励企业在国(境)外设立生产基地、营销网络和参加国际展会。发展跨境电子商务、市场采购等新业态，跨境电子贸易进出口 13.1亿元。全年商品出口总值727.15亿美元，增长15.8%。实际使用外资51.07亿美元，增长6.3%。对外投资31亿美元，增长 70.7%。

　　二是深化重点领域改革，激发市场活力和内生动力。围绕破解制约经济社会发展深层次矛盾和问题，把改革作为根本之策，全面展开13方面226项改革事项。

　　加快行政领域改革。从政府自身改起，大幅度简政放权。动态调整99项行政审批、备案事项，公布10个部门权责清单，推进居民办证制度改革，95.7%的行政审批事项可网上办理。开展政府职能转变和机构改革、事业单位分类改革，推进行政区划调整。全面推行工商登记制度改革，取消外商投资企业联合年检，加强事中事后监管，新设立市场主体增长28%，注册资本增长108.7%。

　　加快财税领域改革。在全国率先建立全口径政府预决算编报体系，将所有财政性资金收支预算向市人民代表大会报告，公开部门“三公”经费预决算，清理整合财政专项资金，完善一般性财政转移支付制度，广州财政透明度排名全国第一。坚持科学理财，增收节支还债建设并重，2013年下半年以来化解存量债务800亿元(其中，市本级552亿元，区248亿元)，存量债务削减26.7%，有效化解系统性政府债务风险。全市一般债务率15.5%(市本级 13.5%)、专项债务率26.4%(市本级25.7%)，债务率处于较低水平。扩大“营改增”试点，取消或降低16项行政事业性收费，通过减税清费为企业减负285.5亿元。

　　加快投融资领域改革。创新金融平台，南沙新区金融改革创新15条政策获批，国际金融城入驻一批金融机构。新设法人金融机构12家，新增上市企业6家、新三板企业36家，股权交易中心挂牌企业突破1000家。与中国人寿、新华人寿合作设立产业投资基金。新增一批社区及农村金融服务站、小额贷款公司、村镇银行和资金互助合作社等普惠金融机构。

　　深化国有企业改革。着手国资国企改革总体设计，将65户直接监管国有企业整合成32户，推动上市公司重组整合，推进企业规范董事会建设，筹建国资发展资本投资和运营平台，加强国资监督管理和廉洁建设。

　　三是注重调整经济结构，提高发展质量和效益。牢固树立“质量第一”的经济增长理念，在优化结构中稳增长，在创新驱动中促转型，为广州长远发展增添后劲。

　　加快产业结构调整。围绕加快推进汽车、精细化工、重大装备、新一代信息技术、生物医药、新材料、新能源与节能环保、商贸会展、金融保险、现代物流等十大重点产业发展，出台促进战略性新兴产业、现代服务业、新业态发展等系列措施，开展专业批发市场转型试点，促进产业升级。服务业比重持续提高，增加值突破万亿元，特别是金融、物流、会展发展较快。工业发展稳中提质，规模以上工业总产值1.82万亿元、增长7.9%，汽车、石化、电子三大支柱产业增长8.8%。战略性新兴产业增加值增长12%。移动互联网、电子商务、智能装备、生物医药、新材料等新业态和先进制造业加速壮大。农业发展平稳，增加值 237.52亿元，增长1.8%。

　　加快科技创新。推进国家创新城市建设，深化科技体制机制改革，启动财政科技经费、科技企业孵化器双倍增计划，推进产学研协同创新，激发了企业创新活力。加快发展中乌巴顿焊接研究院、华南干细胞与再生医学研究中心、伯明翰大学广州中心等新型研发机构，广州知识产权法院挂牌运作，广州超算中心“天河二号”运算速度连续4次全球第一名。现有国家级实验室28家、工程中心18家、质检中心10家。推进“智慧城市”建设，吸引信息产业总部落户，入选“宽带中国”示范城市。规模以上工业高新技术产品产值8001.36亿元，增长9.1%。新增发明专利申请量14589件、授权量4590件。

　　四是加强城市建设管理，提升国家中心城市功能和品质。围绕增强城市综合承载力，加大基础设施建设力度，强化环境治理，完善管理体制，提高综合竞争力和国际影响力。

　　加强城市基础设施建设。推进交通枢纽建设，加快白云机场扩建、南沙港三期、国铁、城轨、地铁、高快速路和市政路桥等一批重大项目建设，机场第三跑道竣工，贵广、南广高铁和广乐高速、肇花高速花都段、新化快速南段建成通车，海珠有轨电车试验段开通，同德围南北高架路、金沙洲大桥拓宽、洲头咀隧道、花城大道东延线及立交等59个路桥项目竣工。新开100条公交线路，新增1000台公交运力，新建50公里公交专用道。推进一批科教文卫养老政法等设施建设，“四馆一园”前期工作基本完成，南越王宫博物馆(一期)和9个儿童公园建成开放，少年儿童图书新馆即将开放，惠爱医院江村院区扩建工程完工，城市功能不断完善。

　　优化城乡生态环境。开展空气治理，实施燃煤机组超洁净排放改造，完成一批重点企业脱硫脱硝工作，淘汰1016座小锅炉和8.7万辆黄标车、老旧车，新增管道燃气用户32.2万户，中心城区基本建成“无燃煤区”，会同周边城市开展联防联控。全年空气优良天数282天，占77.5%，增加22 天，PM2.5年均浓度下降7.5%。开展水环境治理，推进市内河涌和广佛跨界河涌整治，新(扩)建一批污水处理设施，城乡自来水改造工程基本完成，北江引水、北部水厂一期抓紧推进，珠江广州河段平均水质为Ⅳ类。开展垃圾治理，启动创建全国生活垃圾分类示范城市工作，推广垃圾定时定点分类投放，完善垃圾分类收运体系，改造56座垃圾压缩站，3个资源热力电厂开工建设，逐步破解“垃圾围城”难题。防治农业面源污染，化肥、农药使用量均减少3%，规模化畜禽养殖场废弃物利用率达85%。开展生态园林建设，建成岭南花园6个、绿道300公里、生态景观林带129公里、生态公益林10万亩、4个湿地公园，完成森林公园和市内公园升级改造，“花城绿城水城”格局基本形成。

　　推进城市更新改造。强化规划引领，推广“三规合一”成果，完成历史文化名城保护规划和867个行政村规划，推进重大平台核心区控规。完善节约集约用地机制，查处闲置土地93宗、434公顷，查处违法用地1228宗、涉及耕地5458亩。开展“三旧”改造，加快成片连片改造，盘活“烂尾楼”35 宗，整治37个城中村安全隐患和448个专业市场管线，城市面貌出现新变化。

　　提升城市综合治理水平。推进社区网格化服务管理，完成314个幸福社区创建工作。加强城管综合执法，提高市容景观管理水平，集中整治乱摆卖和占道经营，拆除违法建设110万平方米、违法户外广告3.1万平方米，查处违法施工2720宗，整治“群租房”2.2万套。推进平安广州建设，开展安全生产隐患专项整治，保持高压打假治劣态势，破获食品药品类犯罪案件768宗，推动家禽“集中屠宰、冷链配送、生鲜上市”，新增1110间学校食堂安装视频监控系统，组建反恐力量，组织“六大专项”、“雷霆扫毒”等整治行动，完善立体化社会治安防控体系，刑事、治安类警情下降8.9%，群众治安满意度持续提高。

　　五是发展社会民生事业，促进社会公平正义。高度重视民生，市本级一般公共预算支出的76.3%用于民生和各项公共事业，公共服务体系不断健全。

　　保障群众基本生活。组织职业培训，应届广州生源高校毕业生就业率93.3%，城镇新增就业27.08万人。完成2998人积分入户。筹建保障房(含租赁补贴)1.56万套、基本建成1.96万套，改造农村泥砖房和危房9000户，着手建设来穗人员公租房。五险参保人数达2649.7万人次，连续8年提高城乡低保标准，实施城乡居民大病医疗保险制度和商业保险医疗救助制度，企业退休人员月人均养老金突破3000元，农转居人员养老金提高9%。改造升级农村敬老院和413个老年人活动站点，动工新建8325张养老床位。

　　大力发展社会事业。推进教育发展，完成第一期学前教育三年行动计划，实施异地中考，实施教育城建设和大学城提升计划，完善民办教育财政差别化扶持机制，9个区成功创建全国义务教育发展基本均衡县。推进文化繁荣，精神文明创建活动蓬勃开展，开展文化遗产普查，启动文物保护专项资金，扶持非国有文物保护，举办系列国际大型文化活动，完成惠民演出、免费门票、广电节目覆盖等惠民实事，基本完成《广州大典》编纂出版，音乐剧《西关小姐》荣获中宣部“五个一工程”奖。发展医疗卫生事业，加强基层医疗卫生机构建设，推广镇村医疗一体化管理，开展区域医疗联合体试点，组织全科医生培训，推行家庭医生契约服务，组织县级公立医院改革。启动应急机制，防控登革热、埃博拉、禽流感等疫情。推进人口计生服务工作，落实单独二孩政策。发展体育事业，举办10余项国际体育赛事，30个小型足球场、36个“一站两点”、公共体育场馆优惠开放等惠民实事全部完成。

　　加强“三农”工作。加快农业基础设施建设，完成21.3万亩高标准基本农田建设，新增2.1万亩标准化菜田。发展现代农业，8个市级现代农业园区建成10.9万亩。深化农村改革，开展土地承包经营权确权登记试点，推进集体建设用地使用权流转，建成集体经济“三资”交易平台。建设27个美丽乡村和3个名镇、41个名村。市财政安排4.85亿元扶贫，新一轮农村扶贫开发取得成效。

　　推进各项事业全面发展。成功举办第二届广州奖及2014中国国际友好城市大会暨广州国际城市创新大会，成立广州-奥克兰-洛杉矶三城经济联盟，广州成为世界大都市协会亚太地区总部所在地并当选首任联合主席。开展经济普查，普法宣传、法律援助、人民调解工作进一步加强，建立少数民族服务体系。推进梅州、清远、湛江帮扶，对口支援新疆喀什、西藏波密、贵州黔南等地区成效明显。国防动员、海防和打私、双拥和优抚安置、宗教、司法行政、妇女儿童、残疾人、慈善、侨务、对台、贸促、口岸、应急、气象、地震、民防、审计、统计、参事、文史、档案、保密、修志、仲裁、红十字会等工作取得新进步。

　　六是加强政府自身建设，增强政府行政效能。实施重大行政决策事项目录、听证目录和专家论证办法，政府决策更加科学透明。坚持以公开为常态、不公开为例外的原则，主动公开政府信息108.65万条、依申请公开事项8939宗，市政府定期组织新闻发布会，开通市政府常务会议数据库网页。整合34个部门和各区的55条热线为“12345”政府服务热线。推进依法行政，起草地方性法规草案5项，制定政府规章13项和规范性文件27项，受理行政复议案件 1337宗。高度重视各级人大代表议案决议、代表建议、政协提案办理工作，全年办理全国、省、市人大代表建议373件和政协提案510件，办理质量不断提高。继续推进2012年2件和2013年1件议案决议的办理落实，加快“珠江黄金岸线”建设和城市废弃物处置利用，深化“村改居”管理体制综合改革。严格落实中央八项规定精神，持续纠正“四风”，清理行政事业单位“小金库”，“三公”经费较上年减少23.4%，实施公共服务廉洁化“双十部门、双百实事”工程。加大廉政建设和反腐败工作力度，一批违法违纪人员受到惩处。广州被《中国法治政府评估报告2014》、《广东省地方政府整体绩效评估报告》评为第一名。

　　各位代表，过去一年的成绩来之不易。这是党中央、国务院和省委、省政府及市委正确领导的结果，是全市人民团结奋斗的结果。我代表广州市人民政府，向全市人民、驻穗部队官兵、各民主党派、人民团体以及社会各界人士表示崇高的敬意!向关心支持广州改革发展的港澳台同胞、海外侨胞、国际友人表示衷心的感谢!

　　我们清醒认识到，我市经济社会发展中还存在不少矛盾和问题：GDP未能完成预期目标，这既有经济下行压力加大的客观原因，也有对经济运行规律把握不够好的主观原因。经济增长亮点少，工业投资规模小，缺少大项目支撑。产业层次不高，金融、总部经济、科技和人才存在明显差距，新兴产业处于初创时期、体量不大，转型升级任务艰巨。劳动力、土地综合成本上升，融资难、融资贵的问题影响实体经济发展。未来几年偿债量较大,潜在风险不容忽视。城市基础设施承载力和市政配套不足，交通拥堵、环境污染、安全生产风险依然突出，大气、水环境治理和垃圾分类处理任重道远。防控登革热等流行性疫病存在薄弱环节，就业、住房、教育、医疗、养老、社保、食品药品安全、城市管理、社会治安等方面群众不满意的地方依然较多。政府职能转变不到位,社会信用体系不健全，腐败问题时有发生，少数公职人员不勤不廉现象依然存在。对这些问题，政府要高度重视，牢记责任使命，增强忧患意识，勇于担当，扎实有效解决问题，不辜负人民群众的厚望。

　　二、2015年工作安排

　　今年，是全面深化改革的关键之年，是全面推进依法治国的开局之年，是全面完成“十二五”规划的收官之年。市委十届六次全会围绕适应经济新常态、实施“一带一路”战略、推进自贸试验区建设、深化泛珠三角合作的新变化新情况新问题，明确了经济社会发展的目标任务，作出全面推进依法治市的决定，承载着全市人民的深切期望。我们要在全球城市体系中找标杆，从国家大战略中找动力，从区域发展中找动力，从全球发展要素配置和国际产业分工中找动力，主动作为，勇闯难关，努力实现我市经济社会发展提质增效。

　　今年政府工作总体要求是：全面贯彻落实党的十八大、十八届三中、四中全会和中央经济工作会议精神，紧紧围绕巩固和提升国家中心城市地位，坚持稳中求进工作总基调，坚持以人为本、可持续发展，主动适应经济发展新常态，全面实施城市创新发展战略，深化改革开放，强化法治建设，突出结构调整，优化生态环境，提升城市品质，改善民生福祉，打造国际商贸中心、国际航运中心、世界文化名城和华南交通枢纽，促进经济平稳健康发展和社会和谐稳定。

　　今年经济社会发展主要预期目标是：地区生产总值增长8%;地方一般公共预算收入增长10%(可比口径);固定资产投资增长14%;社会消费品零售总额增长10.5%;商品出口总值增长10%;万元生产总值能耗下降2.7%以上，化学需氧量、氨氮、二氧化硫和氮氧化物排放总量分别下降0.2%、 1%、1%和2%;城乡居民收入增长与经济增长基本同步;城市居民消费价格涨幅控制在3%左右。重点抓好五个方面工作：

　　(一)深入推进改革创新。改革创新是广州经济快速发展的制胜法宝。要围绕激发市场活力，以经济改革为抓手，以城市创新为动力，以克服短板为突破口，培育新的经济增长点。

　　深化重点领域改革。全力推进南沙新区和自贸试验区建设，落实外商投资负面清单，争取启运港退税、汽车整车转口保税、汽车平行进口、跨境人民币贷款、离岸数据服务等改革试点，复制上海自贸试验区海关监管和检验检疫制度创新措施，建立知识产权保护机制，形成与国际投资贸易通行规则相衔接的基本制度框架。继续简政放权，再调整一批行政审批、备案事项，实施权责清单，推行审批事项联网通办，完成政府职能转变、机构改革和区划调整。加快推进价格改革，推行“三证合一”工商登记制度，加强事中事后监管，完善社会信用体系。深化财税改革，贯彻落实新预算法，推进政府事权和支出责任相适应制度改革，探索建立跨年度预算平衡机制和实施中期财政规划，完善财政预算绩效管理和政府购买服务改革，完成“营改增”扩面任务，稳步推进税制改革，完善政府性债务管理和防范化解债务风险体系。深化投融资改革，出台企业投资管理体制改革意见，推进企业上市“双百工程”，鼓励民间资本设立金融机构，加快发展创业及股权投资市场。深化国企改革，实施“清单管理”国资监管模式，组建国有资本投资和运营公司，强化资本运作和市场化选人用人机制，发展混合所有制经济，完善公司法人治理结构，激发国有企业活力。

　　推进产业创新发展。坚持先进制造业和现代服务业双轮驱动，加快高端高质高新产业发展，构建集群集聚集约产业体系，做大做强做优十大重点产业，打造产业高地。促进制造业高端化，发展电动汽车，培育轨道交通龙头企业，建设工业机器人及智能装备基地、再生医学等一批高端项目，加快航空和海洋工程装备发展。促进战略性新兴产业发展，建设移动互联网创新产业基地、国际生物岛、国际健康产业城、国际创新城、全生物降解塑料等重大园区和项目，重点发展手机游戏、可穿戴电子产品、高端医疗器械、高端金属材料、高性能高分子材料等产业。促进新业态发展，加快建设新业态总部经济产业园、汽车服务业产业园、融资租赁产业园等15个示范园区，发展工业机器人、电子商务、科技服务、现代物流、工业设计、文化创意、3D打印、大数据应用、卫星导航、移动互联网等新业态。促进传统产业优化提升，推进传统制造业企业技术改造，基本完成“退二进三”任务，推进全国旅游综合改革试点，推动专业批发市场向展贸化、信息化、标准化发展。

　　大力发展科技、金融、总部经济。实施创新驱动战略。继续推进财政科技经费和科技企业孵化器双倍增计划，以“一区多园”模式统筹科技园区发展，申报国家自主创新示范区和国家全面创新改革试点。加大科技成果转化力度，组建一批产学研协同创新联盟，推进科技服务业发展，引进和共建新型研发机构，加快广州超算中心应用推广，实施羊城创新创业人才支持计划，推进粤港澳人才合作示范区和人才资源服务产业园建设，增强自主创新能力。建设知识产权枢纽城市，设立知识产权交易中心，推进国家版权贸易基地建设。推进金融创新发展，完善金融机构主体体系，争取新设一批新型金融机构，重点建设广州国际金融城和南沙现代金融服务区，举办第四届中国(广州)国际金融交易·博览会，出台互联网金融、普惠金融扶持政策，探索建立互联网金融征信体系，积极开展跨境人民币贷款、小额贷款保证保险等创新业务，促进新型金融服务业态的集聚发展。推进总部经济发展，加大总部企业引进力度，实施总部招商行动计划，建立总部企业协调服务机制，加紧在建总部项目建设，吸引航空、会展、电子商务、互联网、智能装备等一批总部项目落户;培育本土总部企业，推进华南地区大中型骨干企业新业态总部基地建设，发展泛珠总部经济区，支持楼宇经济提升发展，促进小微企业健康发展，推动国有企业和老字号企业兼并重组，打造“企业航母”。

　　(二)促进经济提质增效。发挥“三驾马车”均衡拉动作用，优化经济发展空间布局，促进经济行稳致远。

　　发挥消费的基础作用。抓住国家推进消费升级的机遇，加快建设国家电子商务示范城市，促进网上购物和农村电商发展，扩大移动互联网、物联网等信息消费，办好广州国际美食节，大力发展养老健康、文化娱乐、体育健身、旅游休闲和时尚消费。着力打造商贸平台，组建国家级珠宝玉石交易所，设立华南有色金属交易中心，开展国内贸易流通体制改革综合试点、物流标准化试点、餐饮业转型试点，强化消费集聚功能。拓展广货市场腹地，积极参与珠江-西江流域经济带、珠三角、泛珠三角合作，推进广佛肇、广清一体发展，开展高铁沿线城市展销活动，继续推行“广货全国行、广货网上行”展贸活动，扩大消费需求。培育消费潜力，落实收入分配制度改革，健全工资增长机制。

　　发挥投资的关键作用。优化投资结构，强化重点项目带动，推进156个基础设施、产业、民生重点项目建设，实现年度投资1180亿元。加大招商引资力度，继续举办“新广州·新商机”招商推介活动，利用广交会、达沃斯分论坛等平台拓展招商渠道。扩大民间投资，推出一批民间投资重点项目，在准公益领域面向民间资本开放，促进投资稳定增长。

　　发挥出口的支撑作用。推动投资便利化，实施外商准入前国民待遇加负面清单管理模式，实行境外投资备案管理，引进国际高端会议展览，建设国际贸易单一窗口，推行关检合作“三个一”、口岸管理“三互”等试点，营造市场化、法治化、国际化营商环境。培育外贸竞争新优势，发展跨境贸易电子商务、保税物流、租赁贸易、市场采购等新业态，发展服务贸易和服务外包。加强与“一带一路”沿线城市合作，鼓励企业赴境外投资和承包工程，参与海外工业园区、经贸合作区建设。扩大先进技术设备、关键零部件和大宗商品进口。推进中以生物产业孵化基地、中新知识城院士专家创新创业园等合作项目，组织开展中欧区域政策合作试点。拓展国际友城交流，深化穗港澳台合作，提高对外开放水平。

　　发挥南沙、黄埔、增城三个国家级开发区的主引擎作用。南沙要用好用足国家战略政策，抓住自贸试验区和国家级新区双重政策优势，谋划高水平对外开放和投资贸易便利化，加快投资、贸易、金融、监管制度创新，加紧实施金融创新15条政策，落实部际联席会议15条支持事项，抓紧推进港口、地铁、快速路等交通基础设施建设，加快发展航运金融、物流和海洋经济，迅速壮大经济体量，建设国际航运中心和物流中心。黄埔要抓住区划调整的契机，以“两城一岛”为核心，抓紧推进中新知识城上升为国家战略，加快电子商务、检验检测、智能装备、港航服务等专业园区建设，发展知识经济，打造知识产权枢纽和知识密集型产业集聚高地。增城要发挥国家级开发区的政策优势，加快牛仔服装等传统产业转型升级，加快低碳总部园、电子五所、健康医疗综合体等重点产业项目建设，引进一批带动力强的龙头企业，发展中小微金融服务区和普惠金融，快速提升经济实力。同时，空港经济区要大力发展跨境电子商务、现代物流、外贸展示、飞机维修制造和租赁等临空产业。

　　(三)提升城市功能品质。城市发展，关系人民群众福祉，关乎城市未来。围绕建设干净整洁平安有序和有文化底蕴、有岭南特色、有开放魅力的现代化生态宜居城市，科学规划，注重长远，打牢基础，重点推进，全面提升城市综合竞争力和国际影响力。

　　强化基础设施建设。大力推进3个空港项目、5个港航项目、9个铁路工程、6条城际轨道、11条地铁、11条高快速路和130个市政道路项目建设，推进白云机场噪音区治理，新建一批公交设施和停车场，抓好与周边6市58个互联互通重点交通项目，提升交通枢纽功能。加快“四馆一园”、海事博物馆、粤剧艺术博物馆、南粤先贤馆、南汉二陵博物馆、华侨博物馆、第三少年宫等文化设施建设。加快基层医疗卫生机构标准化建设和市属三级医院建设，加快政法设施建设，推进老人院、日间托老机构等养老设施建设，完善城市服务功能。

　　加快城市更新改造。扩大“三规合一”成果应用，加快重大项目规划审批和用地保障，加大土地收储力度，划定城市开发边界、永久基本农田、生态保护红线。加快更新改造步伐，完善城市更新政策，体现城市岭南文化特色，推进同德围、金沙洲、罗冲围、国际金融城、大坦沙岛、广钢新城、广纸片区、鱼珠旧城、海珠生态城等成片连片改造项目，支持历史街区活化利用，为城市发展腾出空间。推进城中村综合治理，开展垃圾处理、管线下地、消防隐患、管道煤气和安全用电整治行动，加强对乱摆卖、“三非”外国人、出租屋集中整治，改善人居环境。开展专业市场整治行动，推进安全隐患整改，改善城市面貌。

　　推进城乡一体发展。提升农业综合生产能力，抓好5个万亩蔬菜生产基地、8个市级现代农业园区建设，建设海鸥岛生态旅游岛，发展生态农业、观光农业，推动农民专业合作社示范社和家庭农场建设。深化农村改革，铺开土地承包经营权确权登记颁证，深化集体经济股份合作制改革，健全土地流转服务机制，推动土地承包经营权抵押担保贷款试点，加快发展农业保险，完善生态保护补偿机制，扩大生态公益林补偿面积，实现生态公益林补偿全覆盖。加强新农村建设，推进美丽乡村、名镇名村、幸福社区创建工作，开展农村土地和环境卫生综合整治，建设新型城镇化示范镇，改善农村人居环境。抓好新一轮市内农村扶贫工作。

　　强化空气和水环境治理。开展清洁能源和火电机组改造，推进“无燃煤街(镇)”创建工作，完成7台燃煤设备超洁净排放改造，完成水泥企业降氮脱硝及除尘改造，淘汰7.77万台黄标车及老旧车，增加管道燃气用户50万户，加强建筑工地和煤、沙码头扬尘管理，完成60%重点挥发性有机物排放企业治理，建立大气污染源清单，提升空气质量。实施南粤水更清行动计划，加强流溪河水源地保护，推进16条广佛跨界河涌整治，新(扩)建16座污水处理厂，城市生活污水处理率达92.5%。新建120个行政村生活污水治理设施，农村生活污水处理率达46%。提高供水保障能力，建设北江引水工程、北部水厂一期及牛路水库工程，完成农村自来水改造，城市饮用水源水质达标率稳定在100%。

　　加强生态环境保护。打造北部旅游、中部休闲、南部滨水生态片区，新增生态景观林带80公里、绿道300公里、森林公园10个，推进生态景观工程，完成儿童公园建设。建立受污染工业场地清单，开展未批先建、无证排污、偷排偷放等整治行动，实施重金属污染综合防治，提高环境质量。创建全国生活垃圾分类示范城市，发展循环经济产业园区。

　　(四)统筹做好民生社会事业。政府的根本目的是让人民群众过上好日子。坚持把改善民生作为工作出发点和落脚点，建机制、补短板、兜底线，不断提高人民生活水平。

　　全面发展社会事业。发展教育事业。实施第二期学前教育三年行动计划和特殊教育提升计划，全面推进义务教育均衡发展，推动国家中小学教育质量综合评价改革实验区工作，完善中小学幼儿园招生制度，推进“区管校用”教师管理体制改革，构建现代职业教育体系，促进民办教育规范特色发展，提高高等教育质量，创建“广东省推进教育现代化先进市”。发展文化事业。大力培育和践行社会主义核心价值观，扎实推进文明城市创建工作，完成全市文化遗产普查，加强抗战遗址等文化遗产保护，开展群众文化活动，组织送戏下基层，加快广播电视“村村通”和无线覆盖升级，举办音乐金钟奖、戏剧梅花奖、纪录片节等大型文化活动，鼓励社会力量参与文化发展。发展卫生事业。继续推进医疗卫生机构建设，深化县级公立医院改革和基层医疗卫生机构综合改革，开展家庭医生契约式服务，推进医联体试点，推行基层首诊、双向转诊，加强住院医师和全科医师培训，鼓励支持社会资本举办医疗机构，做好传染性疾病防控和应急救治。加强人口计生服务工作。发展体育事业。举办大型国际体育赛事，开展群众性体育活动。加强城市民族工作，促进宗教关系和谐，完善公共气象服务，扩大法律援助覆盖面，推进“六五”普法，深化质量强市创建工作，实施标准化战略，开展援疆、援藏、梅州、清远等扶贫工作和全国“双拥模范城”创建工作。继续做好国防动员、海防、打私、侨务、统计、对台、应急、地震、民防、参事、文史、档案、保密、修志、慈善等工作。

　　提高社会保障水平。提高困难群众住房保障水平，在来穗人员和城市公共服务人员公租房建设上有所突破，构建健全有序的住房保障体系。保障失业人员、老龄人口、低收入困难家庭等的基本生活。完善促进就业创业机制，开展高校毕业生、就业困难群体、农村劳动力就业帮扶，保持就业稳定。扩大社保覆盖面，提高企业退休人员、农转居人员和城乡居民养老保险待遇，建立工伤保险待遇调节机制，制定残疾人医疗保险、养老保险办法，构建普惠型社会保险体系。推进机关事业单位养老保险制度改革。推进全国养老服务业综合试点，鼓励社会力量参与养老服务，建设居家养老指导服务平台。实现殡葬基本服务均等化。

　　推进平安广州建设。严厉打击制售假违法行为，营造规范、公平、有序的市场环境。开展食品药品安全整治和社会共治，建设“食得放心”城市。落实安全生产责任，组织重点领域安全专项整治，提升安全生产事故应急救援能力。强化城市精细化管理，完善城市管理综合执法体制，维护市容环境秩序，整治“六乱”黑点，查处违法户外广告，重点打击违法建设，加强“五类车”治理。实施人民调解、行政调解、司法调解联动，培育司法社工机构，推进矛盾纠纷排查化解，深化社区矫正工作。防范和打击涉恐涉暴违法犯罪，加强互联网安全管理，加大金融和通讯诈骗犯罪打击力度，动态监管重点地区和场所治安形势，加强地铁公交等重点行业技防物防建设，提升群体性事件应急处置水平，维护社会和谐稳定。

　　继续办好十件民生实事。一是加强食品安全监管。对本地食品生产企业和食品重点经营主体(超市、食品批发市场)实施100%监督抽检，对食品品种(28大类加工产品、6大类食用农产品)实施100%监督抽检，累计抽检不少于23000批次。加大对农贸市场、食杂店等经营主体的日常巡查、监督抽检力度。二是促进和稳定就业。举办300场次就业专场招聘会，资助劳动力技能晋升培训5万人，实现广州生源应届高校毕业生就业率达90%以上，全市城镇新增就业人数20万以上，帮扶16万名城镇登记失业人员实现再就业，城镇登记失业率控制在3.5%以内。三是完善社区居家养老服务设施网络。新建50个日间托老机构，实现全市所有街镇建有日间托老机构。四是提高特殊人群救助水平。城乡低保标准提高到不低于650元，实现城乡低保标准一体化;从7月1日起，农村五保对象供养标准100%达到当地农村上年度人均收入水平。五是加强基层慢性病防治服务。为60万名高血压患者和17万名糖尿病患者免费提供首诊测血压、建立健康档案、随访干预等健康管理服务。基本公共卫生服务经费从每人每年40元提高到50元。六是加强城中村整治。推进城中村用电改造，缓解78个频繁停电城中村的用电难问题。新增城中村视频监控摄像头9000个，提高城中村的视频覆盖率。七是加强公共交通体系建设。新增50公里公交专用道，提高公交车快速通行能力;优化调整及新增公交线路50条，投放1100台清洁能源公交车辆。增加交通信号自动化控制路口40个，实现全市80%以上的路口自动化管理; 加强对自动化设备的检测维护，确保设备完好率达95%;优化芳村大道等36条“绿波带”交通信号灯设置，使交通更加顺畅。八是加强住房保障。新开工建设、筹集保障性住房1.37万套，新增发放住房租赁补贴1800户。扩大户籍家庭住房保障覆盖面，将户籍家庭住房保障收入线准入标准从年人均可支配收入 20663元调整至29434元。住房租赁补贴标准从每平方米20元提高到25元。九是提高生活垃圾无害化处理能力。全面实施垃圾分类管理，继续推广生活垃圾定时定点分类投放模式;全力推进全市大型垃圾处理设施建设，新增无害化处理能力3000吨/日。十是加强社区文化康乐设施建设。建设34个社区小型足球场。推进全市12个街镇综合性文化服务中心试点建设。

　　(五)大力推进法治政府建设。法律的生命力在于实施。把政府工作纳入法治轨道，用法治思维和法治方式履行职责，加快建设法治政府、诚信政府、廉洁政府、节俭政府。

　　坚持依法行政。出台加强法治政府建设规划，加强经济、民生、社会、生态等方面的制度建设，健全地方法治体系。认真执行市人大及其常委会各项决议和决定，切实做好人大代表建议和政协提案办理工作，定期通报办理情况。推行行政综合执法，理顺市、区、镇(街)行政管理权限。加强行政执法监督，自觉接受人大、政协和社会的监督。

　　深入推进政务公开。推进依法、科学、民主决策，实行重大行政决策合法性审查，健全政府法律顾问制度，加强智库建设，完善公咨委制度，扩大公众对政府决策的有序参与。落实重大决策终身责任追究和倒查机制，进一步扩大行政权力公开范围，公开权力运行流程，公开“三公”经费，增强权力运行透明度。扩大网上办事大厅与实体政务大厅职能范围，打造阳光政府。

　　创新社会服务管理。注重运用法治方式，健全村(居)务公开，发挥社会组织参与社会治理的作用，完善多元主体治理体系。重点推行“五个一”治理模式。“一卡”，全面推行市民卡，拓宽服务事项，为群众提供“一卡式”社会事务服务。“一号”，进一步整合非紧急类政府服务专线，完善“12345”政府服务热线功能，畅通政府服务群众的桥梁。“一格”，全面推行社区网格化服务管理，完善五级联动、三级指挥体制，更好地联系服务群众。“一网”，完善网上办事大厅，推进网上大厅、实体大厅“五统一”。“一窗”，实行全市上下所有行政审批、公共服务事项“前台综合受理、后台分类审批、统一窗口出件”，提高审批效率。

　　建设廉洁政府。坚决贯彻中央八项规定精神等系列要求，加强廉政制度建设，完善作风监督举报平台，防止“四风”问题反弹。加强公务员队伍建设，全面提高公务员素质。加强行政问责和执法监察，强化经济责任审计。加大预防和惩治腐败工作，重点查处十八大后不收敛、不收手，问题线索反映集中、群众反映强烈，现在重要岗位且可能提拔使用的领导干部;纠正“四风”，重点查处中央八项规定出台以后、群众路线教育实践活动开展以来仍然顶风违纪的行为;严格落实 “一岗双责”，强化权力监督，提高政府公信力。

　　各位代表、同志们，人民赋予重托，奋斗创造未来。让我们紧密团结在以习近平同志为总书记的党中央周围，在市委的坚强领导下，为实现广州人民的期待和梦想而努力工作。
